# 704. Antibiotic Use Audit in Hospital-onset Clostridioides difficile (HO-CDI) Cases in a Tertiary Medical Center in New York City

**DOI:** 10.1093/ofid/ofad500.766

**Published:** 2023-11-27

**Authors:** Neeraja Swaminathan, Philip Yune, Kelsie Cowman, Gregory D Weston, Priya Nori, Rachel Bartash, Terrence D McSweeney, Yi Guo

**Affiliations:** University of Utah, Salt Lake City, Utah; Montefiore Medical Center, Bronx, New York; Montefiore Medical Center, Bronx, New York; Montefiore Medical Center and Albert Einstein College of Medicine, Bronx, NY; Montefiore Health System, Bronx, NY; Montefiore Medical Center, Bronx, New York; Montefiore Medical Center, Bronx, New York; Montefiore Medical Center, Bronx, New York

## Abstract

**Background:**

Hospital-onset Clostridioides difficile infection (HO-CDI) is a nosocomial infection that adversely impacts length of stay, morbidity, and mortality, and contributes to 6 billion dollars in U.S. healthcare expenditures. Our aim was to identify and categorize antibiotic prescribing patterns amongst patients with HO-CDI at our institution and design targeted interventions based on these findings.

**Methods:**

A physician-assigned review of electronic medical records for all laboratory-confirmed cases of HO-CDI was conducted at Montefiore Medical Center Moses Campus (Bronx, NY) from July 2022 to March 2023. HO-CDI was defined using the CDC’s NHSN definition as a positive stool test for Clostridium difficile occurring after 3 days of hospital admission. Our hospital follows a 2-step testing algorithm: a positive test occurs when both glutamate dehydrogenase (GDH) and C. difficile toxin are detected. If there is discordance at this stage, a confirmatory polymerase chain reaction (PCR) test is performed. Formed stool specimens are rejected and Montefiore has institutional guidance for appropriate C. difficile testing. Antimicrobial choice, indication, and duration were reviewed. We characterized the inappropriateness of antibiotic use into 3 non-mutually exclusive categories (inappropriate indication, spectrum of activity, or duration).

**Results:**

77 HO-CDI cases were identified from 15 inpatient services encompassing medical, surgical, and intensive care services. Exposure to one or more antibiotics within 30 days prior to CDI diagnosis was seen in 88% of cases (n=68). Of these, 16 (23.5%) had inappropriate antibiotic use (Table 1). Antibiotics were used for longer than clinically indicated in 11 (69%) cases. Inappropriately broad-spectrum antibiotics were used in 9 (56%) cases. Antibiotic use was deemed unnecessary in 4 (25%) cases (Figure 1). The most frequently used antibiotics were piperacillin/tazobactam in 8 (50%) cases, followed by ceftriaxone in 5 (31%) cases, and meropenem in 2 (12.5%) cases (Figure 2).

**Figure:**
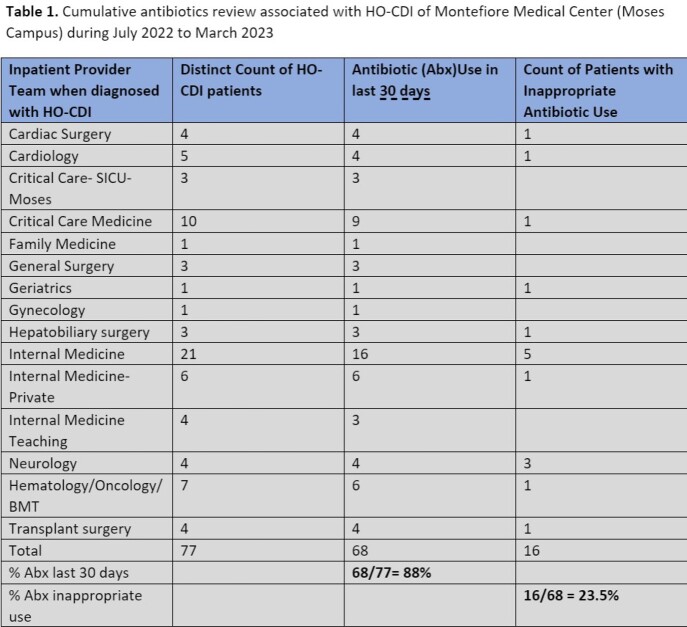

**Figure:**
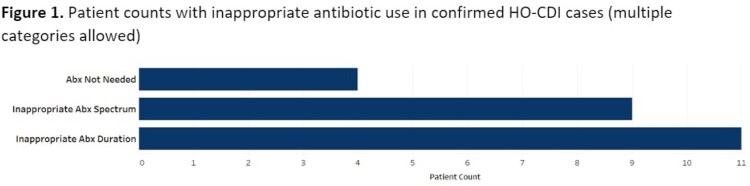

**Figure:**
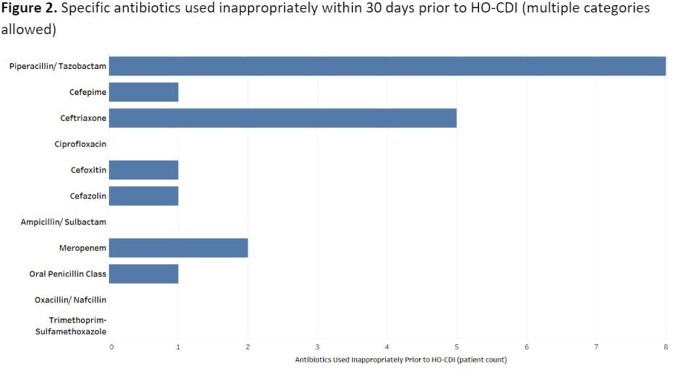

**Conclusion:**

Targeting inappropriate antibiotics is an important strategy to reduce HO-CDI. We intend to follow-up with directed provider feedback and education and heightened antibiotic stewardship efforts.

**Disclosures:**

**All Authors**: No reported disclosures

